# BMPR2 inhibition induced apoptosis and autophagy via destabilization of XIAP in human chondrosarcoma cells

**DOI:** 10.1038/cddis.2014.540

**Published:** 2014-12-11

**Authors:** G Jiao, W Guo, T Ren, Q Lu, Y Sun, W Liang, C Ren, K Yang, K Sun

**Affiliations:** 1 Musculoskeletal Tumor Center, Peking University People's Hospital, Beijing 100044, People's Republic of China; 2 Department of Pathology, Peking University People's Hospital, Beijing 100044, People's Republic of China

## Abstract

Bone morphogenetic proteins (BMPs) are multifunctional proteins, and their receptors (BMPRs) have crucial roles in the process of signaling. However, their function in cancer is somewhat inconsistent. It has been demonstrated that more prevalent expression of bone morphogenetic protein receptor 2 (BMPR2) has been detected in dedifferentiated chondrosarcomas than conventional chondrosarcomas. Here, we find that BMPR2 inhibition induces apoptosis and autophagy of chondrosarcoma. We found that BMPR2 expression was correlated with the clinicopathological features of chondrosarcomas, and could predict the treatment outcome. Knockdown of BMPR2 by small interfering RNA results in growth inhibition in chondrosarcoma cells. Silencing BMPR2 promoted G2/M cell cycle arrest, induced chondrosarcoma cell apoptosis through caspase-3-dependent pathway via repression of X-linked inhibitor of apoptosis protein (XIAP) and induced autophagy of chondrosarcoma cells via XIAP-Mdm2-p53 pathway. Inhibition of autophagy induced by BMPR2 small interfering RNA (siBMPR2) sensitized chondrosarcoma cells to siBMPR2-induced apoptotic cell death, suggesting that autophagy has a protective role for chondrosarcoma cells in context of siBMPR2-induced apoptotic cell death. *In vivo* tumorigenicity assay in mice indicated that inhibition of BMPR2 reduced tumor growth. Taken together, our results suggest that BMPR2 has a significant role in the tumorigenesis of chondrosarcoma, and could be an important prognostic marker for chondrosarcoma. BMPR2 inhibition could eventually provide a promising therapy for chondrosarcoma treatment.

Chondrosarcoma, a malignant tumor of cartilaginous origin, is the second most common primary bone cancer.^1, 2, 3^ For majority of these patients, surgical resection remains the primary therapy of choice; however, there are a large number of inoperable chondrosarcomas located in the pelvic and spinal region. And, usually adjuvant treatments such as chemotherapy and proton beam radiation also do not improve the treatment outcome. Hence, patients with chondrosarcoma continue to have poor prognosis.^4^ Therefore, there is an urgent need to explore a novel therapy that can deal with chondrosarcoma.

Bone morphogenetic proteins (BMPs) is a large family of growth factors that belongs to tumor growth factor-*β* superfamily. They have crucial roles in regulating a wide range of developmental functions, such as pattern formation, differentiation, proliferation, migration, death of cells and so on.^5^ When BMP ligands bind to type 1 and 2 BMP receptors (BMPR1 and BMPR2), BMP signaling is initiated. On activation of the receptor complex by cytokines, the type 1 receptor results in phosphorylation of Smad proteins 1/5/8, which in turn translocate to the nucleus with the common partner Smad4 and then modulate the transcription of BMP-responsive genes.

Although the role of BMPs in the development and formation of bone is well known, their function in cancer is somewhat inconsistent.^6^ On the one hand, it has been shown that BMPs have tumor-suppressive function in carcinomas, particularly in colon cancers in which mutations in BMPR1a and Smad4 are prevalent.^7, 8^ It has been shown that loss of BMPR2 in the stroma of the colon results in epithelial growth as well as polyp formation,^9^ and also the loss of BMPR2 correlates with poor prognosis in prostate cancer patients.^10^ A similar study on human bladder transitional cell carcinoma tissues has shown a frequent loss in the expression of BMPR2.^11^ On the other hand, BMP signaling has also played tumor-promoting roles in some other cancer. Recent data have shown that overexpression of BMPs (especially BMP4 and BMP7) correlates with advanced human breast cancer.^12, 13^ It has also been demonstrated that inhibition of BMPR2 suppresses growth and viability of breast cancer cells.^14^ Furthermore, in our previous study, more prevalent expression of BMPR2 was found in dedifferentiated chondrosarcomas than conventional chondrosarcomas.^15^ However, the mechanisms of BMPR2 in regulating tumor growth and progression remain unclear.

In the current study, we investigated the prognostic value of BMPR2 expression in 78 chondrosarcomas patient, and the effects of BMPR2 small interfering RNA (siBMPR2) on human chondrosarcoma cell lines HCS-2/8 and SW1353. Our results showed that cell growth and cell cycle progression was suppressed after silencing the expression of BMPR2, and it induced apoptosis as well as autophagy *in vitro* and *in vivo*.

## Results

### BMPR2 expression is correlated with clinicopathological features of chondrosarcomas, and predicts treatment outcome

**Figure 1:**
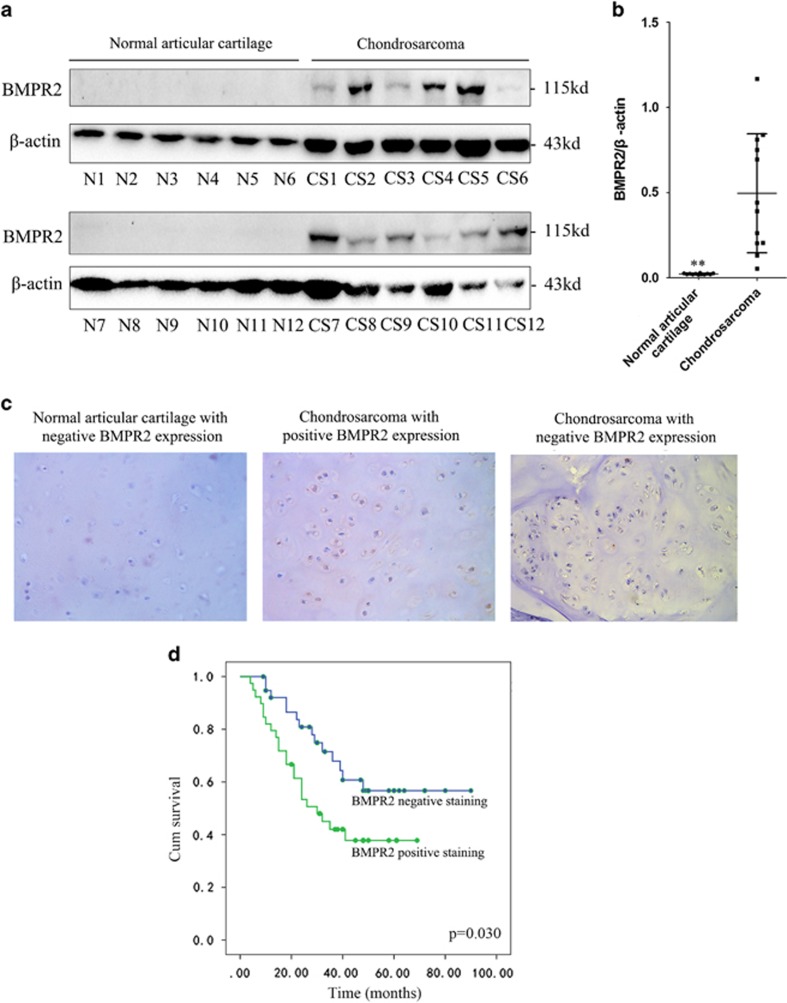
BMPR2 expression is correlated with clinicopathological features of chondrosarcomas, and predicts treatment outcome. (**a**) Western blot analysis showed that BMPR2 was expressed in chondrosarcomas but not in the normal articular cartilage tissues. (**b**) Average expression levels of BMPR2 of normal articular cartilage and chondrosarcoma tissues. Data are presented as mean±S.D. (*n*=12). **P<0.001. (**c**) The expression of BMPR2 in a cohort of 78 human chondrosarcoma specimens was determined by IHC staining of BMPR2. Representative images of BMPR2 IHC staining in normal and chondrosarcoma specimens are shown. (**d**) Kaplan–Meier curves for relapse-free survival in chondrosarcoma patients with or without positive BMPR2 staining. Positive BMPR2 staining (>10% cells stained with BMPR2) in chondrosarcoma specimens is significantly associated with a poorer relapse-free survival (*P*=0.030)

Western blot analyses were performed on 12 samples taken from the normal articular cartilage and chondrosarcoma tissues to investigate the expression of BMPR2 and clinical importance of BMPR2 in chondrosarcomas, respectively. Western blot analysis showed that BMPR2 was expressed in chondrosarcomas but not in the normal articular cartilage tissues (Figures 1a and b). These results indicated that BMPR2 might have tumor-promoting roles in chondrosarcomas. Furthermore, whether BMPR2 expression levels might serve as a biomarker for treatment outcome was evaluated, for that a cohort of 78 chondrosarcoma patients was included in the current study. The BMPR2 expression levels were assessed by using immunohistochemistry (IHC) staining. A total of 50.0% of tumor samples were positive for BMPR2 staining. Representative BMPR2-positive and -negative staining images were shown in Figure 1c. The correlation between BMPR2 expression and the clinicopathological parameters of chondrosarcoma patients was analyzed. As summarized in Table 1, BMPR2 expression was detected as low-grade chondrosarcoma in 7 of 25 patients (grade I), high-grade chondrosarcomas in 14 out of 30 patients (grade I+II) and dedifferentiated chondrosarcomas in 18 of 23 patients. BMPR2 level significantly increased with the advancement of histological grade (*P*=0.002). In addition, by using Kaplan–Meier survival analysis (*P*=0.030), it was found that expression levels of BMPR2 were related with disease-free survival of chondrosarcoma patients. The prognosis analysis showed that the rate of survival in chondrosarcoma patients was poor in case of higher expression levels of BMPR2 (Figure 1d), indicating that BMPR2 could be a prognostic marker for poor prognosis of chondrosarcomas.

### Knockdown of BMPR2 by siRNA results in the inhibitory growth of chondrosarcoma cells

**Figure 2:**
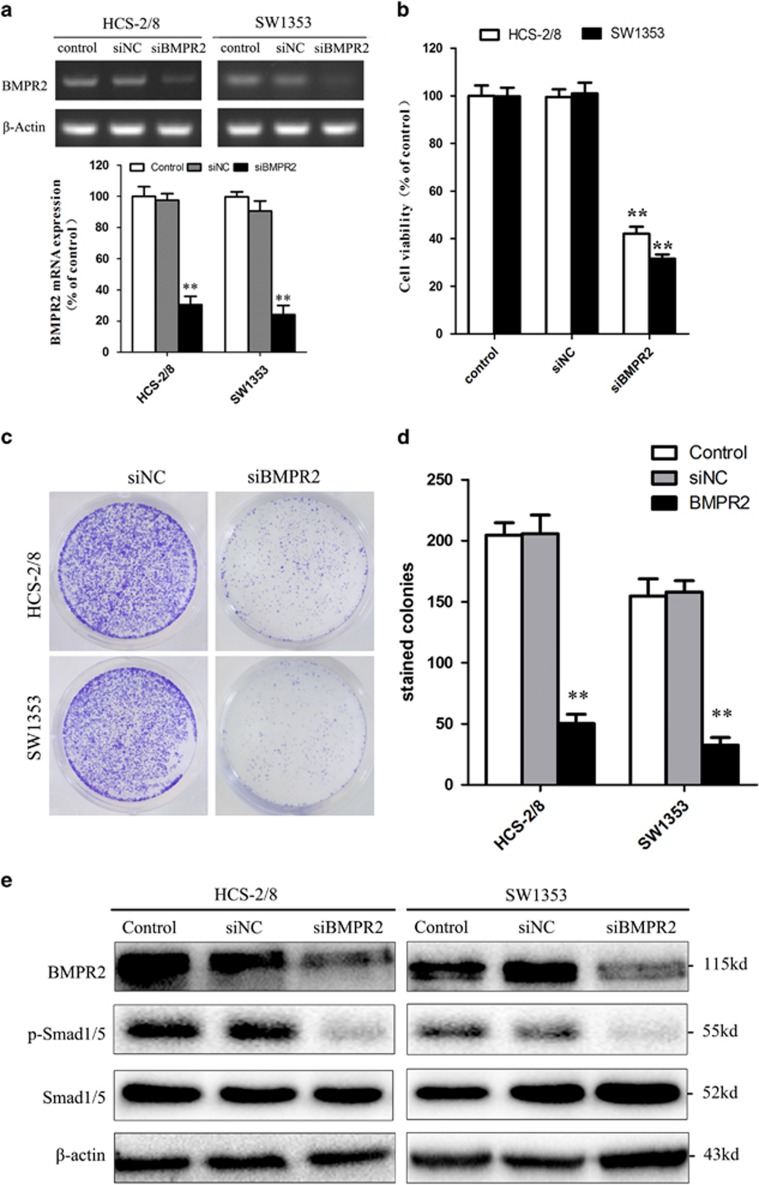
Knockdown of BMPR2 by siRNA results in the inhibitory growth of chondrosarcoma cells. (**a**) Expression of mRNA levels of BMPR2 in HCS-2/8 and SW1353 cells were significantly repressed at 48 h after BMPR2 siRNA transfection. The siBMPR2 transfection efficiency was measured by RT-PCR. (**b**) The chondrosarcoma cell viabilities decreased sharply when treated with siBMPR2 for 48 h, as assayed by MTS. (**c** and **d**) Suppression of BMPR2 significantly decreased capacities of colony formation of HCS-2/8 and SW1353 cells, as analyzed by colony formation assay. (**e**) Expression of protein levels of BMPR2 in HCS-2/8 and SW1353 cells were suppressed significantly at 48 h after siBMPR2 transfection. Inhibition of BMPR2 resulted in the dephosphorylation of Smad1/5, as assayed by western blot analysis. Data are presented as mean±S.D. (*n*=3). **P<0.001

As BMPR2 is expressed in chondrosarcoma but not in normal articular cartilage tissues, it was interesting to research whether inhibition of BMPR2 would kill human chondrosarcoma cell lines (HCS-2/8 and SW1353). Small interfering RNA (siRNA) was used to knock down BMPR2 to investigate the effect of BMPR2 on maintenance of cell viability in chondrosarcoma cell lines. Our results revealed that after 48 h of BMPR2 siRNA transfection, the expression of mRNA and protein levels of BMPR2 in HCS-2/8 and SW1353 cells were suppressed significantly (Figures 2a and e). The cell viabilities (detected by MTS assay) decreased sharply after the siBMPR2 transfection and the resultant values in HCS-2/8 and SW1353 cells were only about 31% and 42% of the values before the knockdown treatment, respectively (Figure 2b). Colony formation assay showed that owing to the inhibition of BMPR2, capacities of colony formation of HCS-2/8 and SW1353 cells significantly decreased (Figures 2c and d). Furthermore, inhibition of BMPR2 resulted in the dephosphorylation of Smad1/5, which sequentially led to the inactivation of BMP signal pathway (Figure 2e). Therefore, repressing BMPR2 inhibited chondrosarcoma cell growth.

### Inhibition of BMPR2 by siRNA promoted G2/M cell cycle arrest

**Figure 3:**
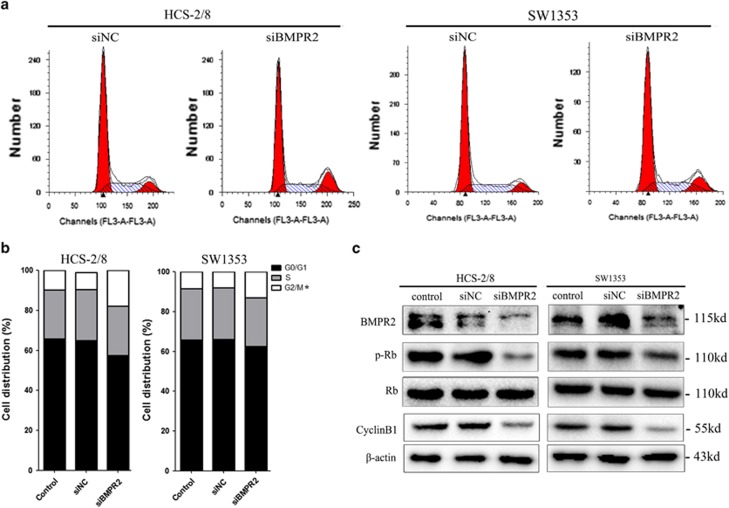
siRNA-mediated knockdown of BMPR2 silencing promoted G2/M cell cycle arrest. (**a**) Cells ware stained with PI following treatment with or without siBMPR2 transfection for 48 h. Cell cycle was analyzed using flow cytometry. (**b**) The bar chart shows the percentage of cells in cell cycle. The BMPR2 siRNA transfection resulted in a significant increase in the proportion of cells in the G2/M phase in HCS-2/8 and SW1353 cells. (**c**) Expression of p-Rb and cyclin B1 were inhibited in chondrosarcoma cell lines when treated with siBMPR2 for 48 h, as assayed by western blot analysis. Representative data from one of three independent experiments are shown in (**a**–**c**)

The inhibition of cell growth could be a result of cell cycle arrest. Therefore, propidium iodide (PI) staining and flow cytometry were used to further determine whether the siBMPR2 altered the distribution of chondrosarcoma cells in different stages of the cell cycle and its progression. The results showed that when the HCS-2/8 and SW1353 cells were transfected with siBMR2 for 48 h, the cell cycles were evidently arrested at the G2/M phase and the proportion of cells at the G2/M stage was increased by 5.09±0.57% and 9.35±1.09%, respectively, when compared with negative control groups (Figures 3a and b). In addition, we found that the expression of p-Rb and cyclin B1, which have been demonstrated to be associated with G2/M stage arrest, were decreased in chondrosarcoma cell lines when treated with siBMPR2 for 48 h (Figure 3c).

### BMPR2 siRNA promoted apoptosis of chondrosarcoma cells via destabilizing of XIAP

**Figure 4:**
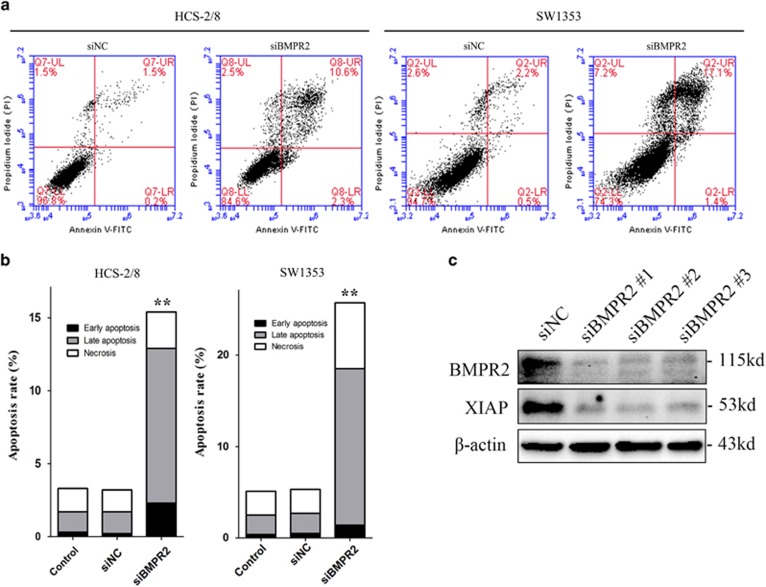

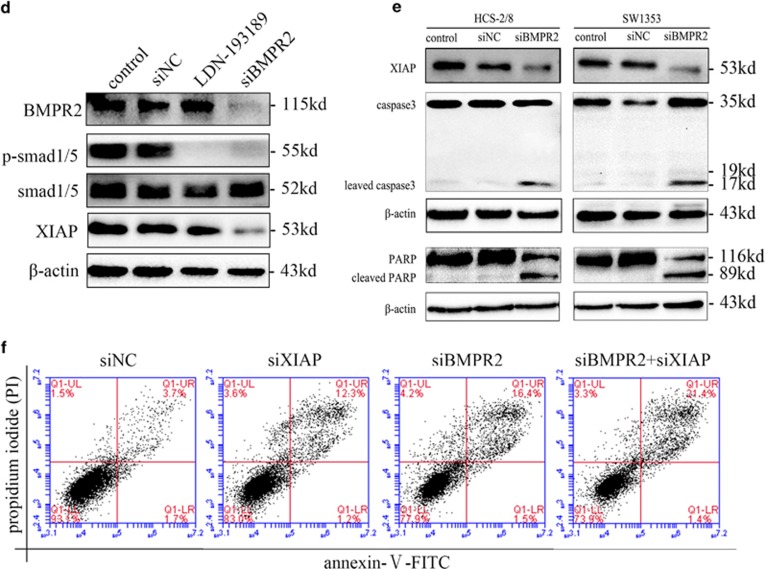
BMPR2 siRNA promoted chondrosarcoma cell apoptosis via destabilizing of XIAP. (**a**) Cells transfected with siBMPR2 were stained with Annexin-V-FITC (20 mg/ml) and PI (20 mg/l). Apoptosis analysis was performed by flow cytometry. (**b**) The bar graph showed a significant increase in the early and late apoptotic index. (**c**) Three BMPR2 siRNA sequences were used to repress BMPR2 in SW1353 cells, and the level of XIAP was sharply reduced when treated with siBMPR2s. (**d**) A BMP signaling inhibitor, LDN-193189, was used to block the BMP/Smads pathway. LDN-193189 did not reduce the level of XIAP, whereas knockdown of BMPR2 did reduce the protein level of XIAP. (**e**) Expression of XIAP decreased, and cleavages of caspase-3 and PARP enhanced in both HCS-2/8 and SW1353 cells when treated with BMPR2 siRNA for 48 h, as assayed by western blot. (**f**) Annexin-V-FITC (20 mg/ml) and PI (20 mg/l) dual staining were performed when SW1353 cells were transfected with siXIAP for 48 h. Apoptosis analysis was performed by flow cytometry. Representative data from one of three independent experiments are shown in (**a** and **c**–**f**)

Chondrosarcoma cells were analyzed by flow cytometry following Annexin-V-FITC and PI dual labeling, to determine whether inhibition of BMPR2 causes cell death by apoptosis. The results revealed that the proportions of early and late apoptotic cells (labeled as LR and UR in Figure 4a, respectively) increased strikingly when treated with siBMPR2 for 48 h, as compared with untreated controls (Figure 4b).

Furthermore, to investigate the mechanism of apoptosis resulted from siBMPR2, we studied the effect of silencing BMPR2 on the expression of X- linked inhibitor of apoptosis protein (XIAP) (a member of inhibitors of apoptosis proteins (IAPs) family, which were originally identified in baculoviruses and exist in organisms from viruses to humans^16^), caspase-3 and poly-ADP-ribose polymerase (PARP). XIAP can inhibit the initiator caspase-9, as well as the effectors caspase-3 and -7. DNA damage or other events that result in induction of apoptosis can lead to ubiquitination and autoubiquitination of XIAP.^17^ However, activation of BMPR2 can stabilize XIAP via preventing ubiquitination and subsequent degradation.^18^ As shown in Figure 4c, in chondrosarcoma cells, silencing BMPR2 by siRNAs led to significantly decreased expression of XIAP.

Although XIAP was recognized as an adaptor for BMPR signaling through the Smad-independent pathway,^18, 19, 20^ LDN-193189, a BMP signaling inhibitor, was used to block the BMP/Smad pathway to determine whether XIAP was loaded in the downstream of Smad-dependent pathway or -independent pathway in chondrosarcoma cells. We found that both LDN-193189 (5 nmol/l) and siBMPR2 reduced the expression of phospho-Smad1/5/8 (p-Smad1/5/8), suggesting significant blockage of BMP/Smad pathway. In addition, we found that siBMPR2 caused a decreased expression of XIAP, whereas LDN-193189 led to no change of expression of XIAP, indicating that XIAP might be located in Smad-independent pathway. Taken together, these results suggest that knockdown of BMPR2 reduced the level of XIAP through a Smad-independent pathway in chondrosarcoma cells.

Furthermore, cleavage of caspase-3, an executioner caspase, had been detected during the execution of the apoptotic cascade. PARP, as a key executor of cell apoptosis, can be cleaved by caspase-3 in response to death-inducing stimuli and the normal PARP function is lost, which irreversibly commits the cell to undergo cell death.^21^ As per our study, it was evident that when HCS-2/8 and SW1353 cells were treated with BMPR2 siRNA for 48 h, both had cleavages of caspase-3 and PARP present in them (Figure 4c).

**Figure 5:**
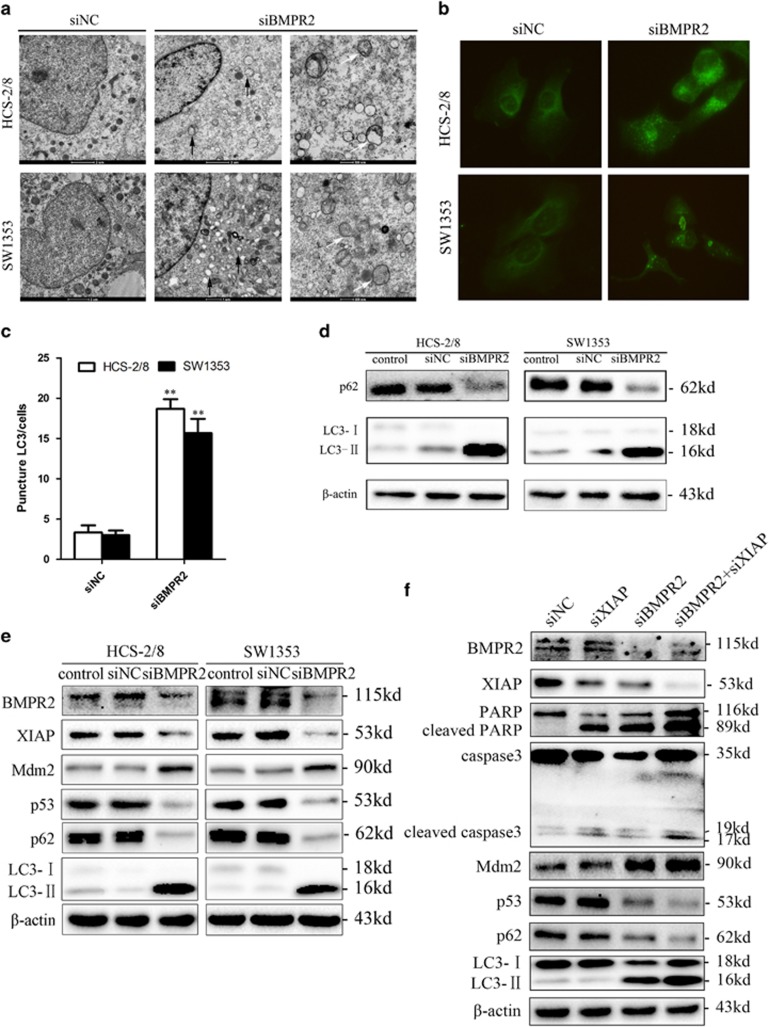
BMPR2 silencing treatment induced autophagy via XIAP-Mdm2-p53 pathway. (**a**) TEM images showing autophagic vacuoles (arrows) observed in siBMPR2-transfected chondrosarcoma cells for 48 h (middle and right). No or few autophagic vacuoles are observed in siNC-transfected cells (left). (**b**) Cells were transfected with BMPR2 siRNA for 48 h, then were incubated with rabbit polyclonal anti-LC3 Ab (1 : 200) overnight at 4 °C and then reacted with anti-rabbit IgG conjugated with Dylight 488 (1 : 400) for 1 h at room temperature. The cells were then visualized using confocal microscopy. (**c**) Quantification of the number of LC3-II punctas in the siNC- and siBMPR2-transfected cells, plotted as LC3-II punctas per cell. Data are presented as mean±S.D. (*n*=3). **P<0.001. The *P*-values were calculated using a two-sided Student's *t*-test. (**d**) Downregulation of BMPR2 induced accumulation of LC3-II and degradation of p62 (a known autophagy substrate) when treated with BMPR2 siRNA for 48 h, as assayed by western blot. (**e**) Silencing BMPR2 resulted in the degradation of XIAP, and subsequently enhancement of Mdm2 led to repression of p53 and eventually induced accumulation of LC3-II and degradation of p62, as assayed by western blot analysis. (**f**) Cleaved caspase-3 and PARP were increased when SW1353 cells were treated with siXIAP for 48 h, whereas p62 and LC3 remained unchanged. When cells were transfected with both siXIAP and siBMPR2, siRNAs significantly increased the expression of Mdm2, LC3-II, cleaved PARP and caspase-3, and decreased the expressions of p53 and p62, compared with when transfected with siBMPR2 alone, as assayed by western blot analysis. Representative data from one of three independent experiments are shown in (**a**, **b**, **d**, **e** and **f**)

To confirm that the apoptosis triggered by siBMPR2 was caused by destabilizing of XIAP, XIAP siRNA was used to repress the expression of XIAP. Flow cytometry following Annexin-V-FITC and PI dual labeling was performed. As shown in Figure 4f, inhibition of both XIAP and BMPR2 increased the number of apoptotic cells compared with the negative control, and downregulating XIAP in the absence of BMPR2 enhanced the number of apoptotic cells compared with downregulating XIAP or BMPR2 alone. In addition, we performed western blotting to detect the expressions of cleaved caspase-3 and PARP. Similarly, we found that downregulation of both BMPR2 and XIAP led to obviously increased cleaved PARP and caspase-3, indicating that inhibition of BMPR2 or XIAP induced apoptosis of chondrosarcoma cells. In addition, siBMPR2 promoted the inhibition of XIAP, and enhanced the expression of cleaved PARP and caspase-3 in SW1353 cells transfected with siXIAP, indicating that when cells were transfected with both siXIAP and siBMPR2, siRNAs significantly accelerated the apoptosis of chondrosarcoma cells as compared with when transfected with siXIAP or siBMPR2 alone (Figure 5f). Jointly, these results demonstrate that apoptosis, mediated by XIAP-caspase-3-PARP, was involved in the response of chondrosarcoma cells to siBMPR2 treatment.

### BMPR2 silencing treatment induced autophagy via XIAP-Mdm2-p53 pathway

Autophagy is a basic catabolic mechanism that has been characterized as an adaptation to stress, which promotes cell survival by ensuring the delivery of metabolic substrates to cells to fulfill their energy demand during stress through a process by which damaged or unnecessary proteins are engulfed in autophagosomes and then digested via the lysosomal pathway; however, autophagy also mediate cellular demise that is called autophagic cell death, depending on the specific circumstances.^22^ To determine whether BMPR2 is involved in the regulation of autophagy, the ultrastructures of autophagy were detected by using transmission electron microscopy (TEM). Figure 5a showed that BMPR2 deficiency caused marked increase in autophagic vesicle formation in HCS-2/8 and SW1353 cells. In addition, to track the conversion of microtubule-associated protein light chain 3 (LC3)-I to LC3-II in autophagosome formation in living cells, immunofluorescence was also used for viewing the expression of LC3-II. Our results showed that LC3-II expression was enhanced in both the cell lines when BMPR2 was inhibited by siRNA (Figures 5b and c). The subsequent western blot analysis also revealed that when BMPR2 was depleted in both the cell lines, the conversion of LC3-I to LC3-II was sharply increased, and p62 (a known autophagy substrate) was significantly degraded (Figure 5d). Collectively, these results elucidate that suppression of BMPR2 using siRNA induces autophagy of chondrosarcoma cells.

Recent evidence has demonstrated that XIAP has been shown to inhibit autophagy via Mdm2 (P53 E3 ubiquitin protein ligase), which is a negative regulator of p53.^23^ To further explore the contribution of BMPR2 in autophagy regulation, we studied the relationship between BMPR2 and XIAP-Mdm2-p53 pathway. Results of western blot analysis assay revealed that silenced expression of BMPR2 leads to reduction of XIAP along with subsequent enhancement of Mdm2, which in turn causes downregulation of p53, which eventually caused autophagy (Figure 5e).

To further confirm the evidence that silencing BMPR2 induced autophagy via XIAP-Mdm2-p53 pathway, XIAP siRNA was used to knock down the XIAP. As shown in Figure 5f, the expressions of Mdm2 and p53 were not changed, and the autophagy-related proteins, such as p62 and LC3-II, remained unchanged when transfected with siXIAP alone, indicating that siXIAP did not induce autophagy in chondrosarcoma cells. However, siXIAP promoted autophagy in chondrosarcoma cells transfected with siBMPR2, and when cells were transfected with both siXIAP and siBMPR2, siRNAs significantly increased the expression of Mdm2 and LC3-II, and decreased the expression of p53 and p62, compared with when transfected with siBMPR2 alone. The results showed that autophagy was enhanced by XIAP knockdown in the absence of BMPR2. Taken together, these outcomes elicited that the autophagy induced by downregulated expression of BMPR2 in chondrosarcoma cells was concerned with XIAP-Mdm2-p53 pathway.

### Inhibition of siBMPR2-induced autophagy sensitized chondrosarcoma cells to siBMPR2-induced apoptotic cell death

**Figure 6:**
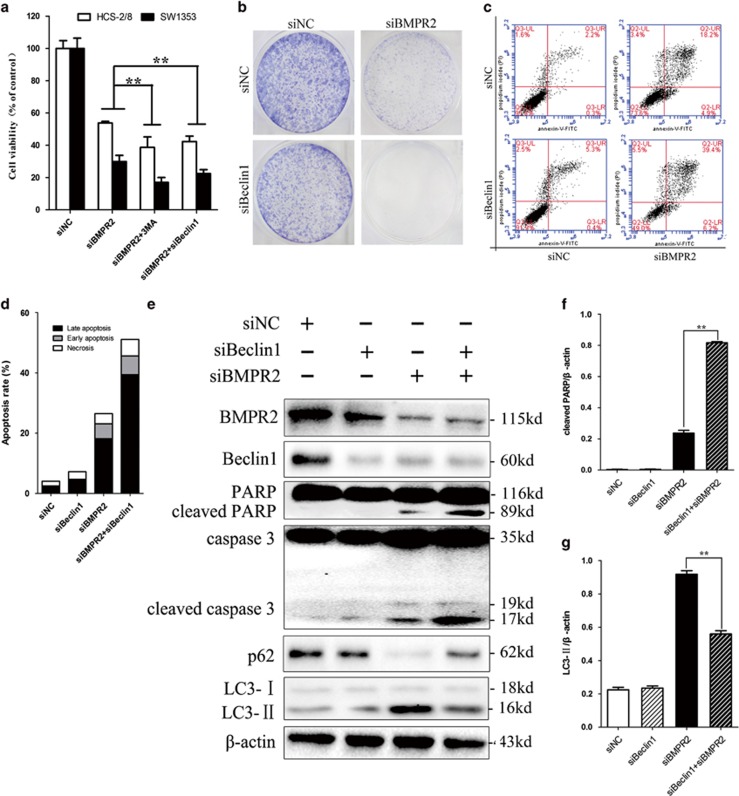
Inhibition of siBMPR2-induced autophagy sensitized chondrosarcoma cells to siBMPR2-induced apoptotic cell death. (**a**) Pretreatment of cells with 3-MA or siBeclin-1 sharply reduced the number of viable siBMPR2-treated cells, as assayed by MTS. (**b**) Colony formation of chondrosarcoma cells was decreased more sharply by Beclin-1 and BMPR2 siRNA transfection than by BMPR2 siRNA. (**c**) SW1353 cells were stained with Annexin-V-FITC (20 mg/ml) and PI (20 mg/l) following siBMPR2 treatment with or without siBeclin-1 transfection for 48 h. Apoptosis was analyzed by flow cytometry. (**d**) Compared with siBMPR2 transfection group, the bar graph showed a significant increase in apoptosis rate in co-transfection of siBMPR2 and siBeclin-1. (**e**) Apoptosis- and autophagy-related proteins were examined by western blot following siBMPR2 transfection with or without siBeclin-1 for 48 h. (**f**) Compared with siBMPR2 transfection group, the bar graph showed a significant increase in cleaved PARP in co-transfection of siBMPR2 and siBeclin-1 (*P*<0.01). (**g**) The bar graph showed a great decrease in LC3-II in co-transfection of siBMPR2 and siBeclin-1 compared with siBMPR2 transfection group (*P*<0.01). Representative data from one of three independent experiments are shown in (**b**–**e**)

Autophagy, depending on the cellular contexts and stimulus, can either suppress or promote tumor cell growth.^24, 25^ As regulation of autophagy may enhance the efficacy of anticancer therapeutics,^26, 27^ we were eager to evaluate whether autophagy induced by siBMPR2 in chondrosarcoma favored cell survival or cell death. Pharmacological approach used for suppressing autophagy (e.g., 3-Methyladenine (3-MA), hydroxychloroquine, bafilomycin A1 or monensin) has been used to elucidate the relationship between autophagy and cell death.^28^ It has been reported that 3-MA is able to block the induction of LC3-II puncta formation.^29^ And, pretreatment of cells with 3-MA sharply reduced the number of viable siBMPR2-treated cells, as assayed by MTS (Figure 6a). siRNA was used to downregulate Beclin-1, a critical autophagic regulator,^30^ to corroborate the cytoprotective effect of autophagy, as pharmacological inhibitors of autophagy may exert autophagy-independent effects. Similarly, siBeclin-1 promoted cell death in chondrosarcoma cells transfected with siBMPR2 (Figure 6a), and when cells were transfected with both siBeclin-1 and siBMPR2, siRNAs significantly decreased capacities of colony formation as compared with when transfected with siBMPR2 alone (Figure 6b). Furthermore, pretreatment of cells with siBeclin-1 enhanced the siBMPR2-induced apoptosis (Figures 6c and d), whereas knockdown of Beclin-1 enhanced cleaved PARP and caspase-3, and reduced LC3-II accumulation (Figures 6e–g; western blot assay), suggesting that autophagy is cytoprotective for siBMPR2-induced apoptotic cell death. Collectively, the results suggest that autophagy inhibition promoted siBMPR2-mediated apoptosis process, which indicates that autophagy has a protective role for chondrosarcoma cells in the context of siBMPR2-induced apoptotic cell death. In addition, these results also demonstrate that inhibiting BMPR2 triggers autophagy in chondrosarcoma, whereas inhibition of autophagy promotes apoptosis, suggesting that autophagy has a cytoprotective role for chondrosarcoma cells in the context of siBMPR2-induced apoptotic cell death.

### BMPR2 inhibition suppressed chondrosarcoma tumor growth *in vivo*

**Figure 7:**
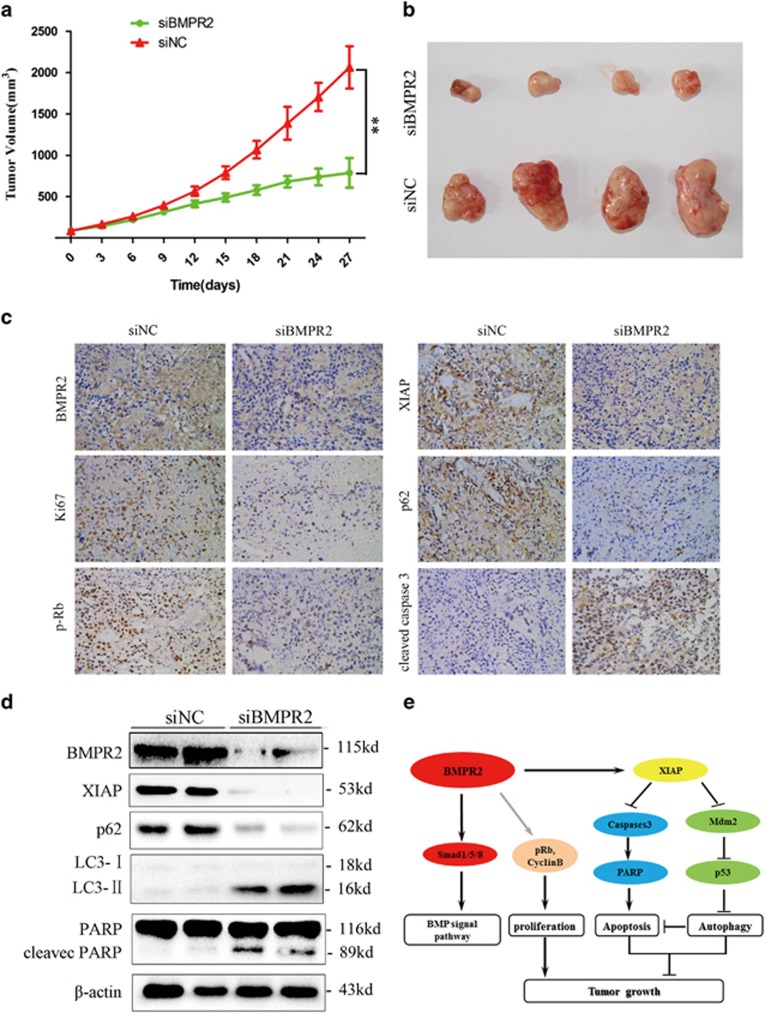
BMPR2 inhibition suppressed chondrosarcoma tumor growth *in vivo.* (**a**) Tumor volume growth curve after intratumor injection of siBMPR2 or siNC. BMPR2 siRNA treatment resulted in significantly decreased tumor growth, compared with the siNC group (Dunnett's *t*-test, *P*<0.01). (**b**) Representative images of xenograft tumors. (**c**) Representative images ( × 400) of IHC analysis of BMPR2, Ki-67, p-Rb, XIAP, cleaved caspase-3 and p62 in tumors. (**d**) Representative western blot analysis elucidating a significant repression of BMPR2 protein level in the BMPR2 siRNA treatment group compared with the siNC group. Changes of XIAP, PARP, P62 and LC3-II proteins followed the same trend as *in vitro*, assayed by western blot analysis. (**e**) Proposed mechanisms responding to BMPR2 siRNA-induced effects in chondrosarcoma cells

To confirm the *in vitro* findings shown above, *in vivo* xenograft models were established. The mouse group injected with siBMPR2 had a lower proliferation rate, and formed evidently smaller tumors than the siRNA negative control (siNC) group, as shown in Figures 7a and b. The tumor volume at the time of death in the siBMPR2 group was 787.27±178.73 mm^3^, which was significantly less than in the siNC group (2063.88±256.39 mm^3^). These data suggest that siBMPR2 reduces tumor volume and growth rate of chondrosarcoma cells *in vivo*.

In IHC and western blotting assay, performed to determine whether BMPR2 expression was suppressed by siBMPR2 *in vivo*, we found that the expression of BMPR2 was largely inhibited by BMPR2 siRNA and later on XIAP, Ki-67, phospho-Rb (p-Rb), caspase-3, PARP, P62 and LC3-II were also detected. Cleaved caspase-3 staining revealed that cleaved caspase-3-positive cells were significantly increased in mice of the siBMPR2 group compared with that of the siNC group, indicating that siBMPR2 enhanced apoptosis (Figures 7c and d). Tumors exhibited decreased p62 IHC, whereas LC3 conversion was enhanced, as assayed by western blot analysis, indicating increased autophagy following siBMPR2 treatment (Figures 7c and d). In addition, IHC using an antibody against Ki-67, a cellular marker for proliferation, and p-Rb, a protein associated with G2/M stage arrest, showed that Ki-67-positive cells and p-Rb-positive cells were both reduced in siBMPR2-treated mice as compared with siNC-treated mice (Figure 7c), demonstrating decreased cell proliferation induced by siBMPR2 administration (Figure 7c).

We also investigated whether siBMPR2 affects the expression of XIAP of human chondrosarcoma cells *in vivo*. IHC and western blot analyses revealed decreased expression of XIAP in tumors from mice treated with siBMPR2, as compared with siNC-treated mice (Figures 7c and d). These results, taken together, indicate that the loss of expression of BMPR2 via siRNA treatment leads to an inhibitory growth of chondrosarcoma.

## Discussion

Results from the current study demonstrated that overexpression of BMPR2 may lead to chondrosarcoma tumorigenesis. Although BMPR2 has been studied in many types of cancers,^9, 10, 11, 14, 31^ the association between BMPR2 and prognosis of chondrosarcoma has not been reported. Our results show that higher BMPR2 expression is associated with higher pathological stage and poorer survival, and also illustrate the antineoplastic effects of siBMPR2 on chondrosarcoma cells *in vitro* and *in vivo*. In brief, our study proposes a novel mechanism, which concluded that G2/M-phase arrest, the cell apoptosis and autophagy are the responsible factors for the antitumor effects of siBMPR2 (Figure 7d).

BMP signaling pathway has great functions on regulating a wide range of developmental fuctions, and BMPR2, as one of BMPRs, has a very critical role in this signaling pathway. Although in certain circumstance BMPR2 may be dispensable for some developmental process, such as the formation of the limb skeleton,^32^ it has important roles in the oncogenesis and development of some malignant tumors.^10, 11, 14, 31^ Voorneveld *et al.*^33^ showed that BMPR2 increased migration and invasion of Smad4-negtive colorectal cancer cells, and the presence of normal BMPR expression in the Smad4-negative colorectal cancer tumors was associated with a poor prognosis. They further illustrated that there was a direct association between LIMK and BMPR2, and Smad4-independent BMP signaling activated the Rho/ROCK/LIMK pathway in colorectal cancer cells. Therefore, BMPR2 also has a vital role in Smad-independent pathway.

IAP family have important functions in the regulation of apoptosis via suppressing activation of caspases and interdicting both mitochondria and death receptor-mediated apoptosis pathways.^16, 34^ Recently, several IAPs, including XIAP, have been shown to modulate autophagy.^23^ XIAP inhibits both apoptosis and autophagy via inhibition of caspase-3 and Mdm2-p53 signaling, respectively.^35^ Besides, it has been reported that inactivation of BMPR2 caused degradation of XIAP and induced apoptosis in mouse embryo fibroblast cells and human umbilical vein smooth muscle cells.^18^ Moreover, radiosensitivity of grade II chondrosarcoma cells was enhanced after silencing XIAP by siRNA.^36^ Intriguingly, our *in vitro* and *in vivo* findings demonstrated that downregulating BMPR2 by siRNA led to the repression of XIAP and induction of apoptosis and autophagy in chondrosarcoma cells.

Apoptosis and autophagy are two vital mechanisms of regulating cell survival and homeostasis.^37^ Involvement of apoptosis, a first known programmed cell death mechanism, in pathogenesis of disease has been well demonstrated.^38^ Autophagy, a critical homeostatic mechanism, was initially considered as a cell survival mechanism as it protected cell through nutrient shortage. Nonetheless, under certain circumstances, autophagy can promote cell survival and avert apoptosis.^22, 39^ Recently, it has been demonstrated that BMP signaling contributed to mediate autophagy in cancer cells.^40^ In the present study, we elucidate that downregulating BMPR2 induces autophagy in chondrosarcoma cells via XIAP-Mdm2-p53 signal pathway. However, inhibition of autophagy induced by siBMPR2 sensitized chondrosarcoma cells to siBMPR2-induced apoptotic cell death, suggesting that autophagy is a prosurvival mechanism rather than a cell death mechanism for chondrosarcoma cells in the context of siBMPR2-induced apoptotic cell death. Although the conditions in which autophagy serve as a prosurvival or prodeath mechanism are not yet definitive, it is supposed that inhibiting autophagy facilitates apoptosis in cancer cells with apoptotic signaling pathways.^41^ In fact, the exact mechanism of siBMPR2-induced autophagy in different kinds of cancers and different stages require further study.

In the current study, we focused on how Smad-independent pathway, specifically XIAP, regulated the growth of chondrosarcoma cells, but the crosstalk between Smad-independent and -dependent pathway has not been well understood. Therefore, we are trying to elucidate the specific mechanism of interaction of these two pathways that coordinate mediate the survival of chondrosarcoma cells. In addition, more research is needed to explore the exact machanism between BMPR2 and cell cycle relative proteins, such as cyclin B1 and p-Rb (Figure 7d).

In conclusion, results from this study demonstrate that BMPR2 has a crucial role in promoting tumorigenesis and growth of chondrosarcoma. The specific mechanisms have been systematically expounded and proved in this paper. Results from this study have the direct potential to develop BMPR2 as an important biomarker for prognosis of chondrosarcoma and a therapeutic target for chondrosarcoma, especially for those with BMPR2 overexpression.

## Materials and Methods

### Patients, tissue samples and follow-up

Twelve fresh and normal human articular cartilage and 12 chondrosarcoma tissue samples were collected under the protocols approved by the ethics committee of Peking University People's Hospital. Informed consents (written in the light of the ethical guidelines) were obtained from all the patients. Seventy-eight formalin-fixed and paraffin-embedded tissue specimens of histopathologically diagnosed chondrosarcoma were obtained from the Department of Pathology and the Musculoskeletal Tumor Center, Peking University People's Hospital (Beijing, China). Tissue samples were collected after operation, sectioned (4 *μ*m thickness) and preserved properly at room temperature until required for the experiment. Clinical and histopathologic information was recorded through a retrospective review of patient records.

### IHC assays for BMPR2

Tissue sections were deparaffinized and antigen was retrieved by antigen retrieval solution. Paraffinized sections were then incubated with mouse polyclonal anti-BMPR2 antibody (dilution: 1 : 100) overnight at 4 °C, followed by a biotinylated secondary antibody staining. Known positive controls were included in each experiment, and negative controls were obtained by staining with nonimmune mouse serum (1 : 100 dilution) in phosphate-buffered saline (PBS) instead of primary antibody. When >10% of tumor cells were positive for BMPR2 staining, the tumor was considered positive for BMPR2 staining. Immunostaining was evaluated by two independent pathologists, who were blinded to clinical characteristics and outcomes.

### RNA extraction and qPCR analysis

Total RNA was isolated from fresh tissue samples and HCS-2/8 and SW1353 cells with Trizol reagent (Life Technologies-Invitrogen, Oakville, ON, Canada), according to the manufacturer's instructions. Then, the purificated RNA was converted to complementary DNA (cDNA) using the First-Strand cDNA Synthesis Kit (Invitrogen) according to the manufacturer's instructions. The cDNA was amplified using Super Script One-Step RT-PCR with the Platinum Taq System (Invitrogen). The primers used for amplification of BMPR2 and *β*-actin transcripts were as follows: BMPR2 forward, 5′-ATCCAGATTATTCTTCCTCCTC-3′ and BMPR2 reverse, 5′-TCACGATGCTGTCAGTATG-3′ *β*-actin was used as an internal control, with forward primer, 5′-ATCAGCAAGCAGGAGTATG-3′ and reverse primer, 5′-GTGTAACGCAACTAAGTCAT-3′. The reactions were performed in a 96-well optical plate (Applied Biosystems, Warrington, UK) for 2 min at 94 °C, followed by 38 cycles of 94 °C  for 45 s, 56 °C for 45 s and 72 °C for 40 s, as described previously.^42^

### Cell culture, cell viability and colony formation assay

Two human chondrosarcoma cell lines SW1353 and HCS-2/8 were used: cell line SW1353, grown in L-15 medium supplemented with 10% FBS and antibiotics, was obtained from ATCC, whereas another cell line HCS-2/8 was maintained in DMEM/F12 medium supplemented with FBS and antibiotics. All experiments were performed during the exponential phase of cell growth. siRNA was transfected into cells against BMPR2 to knock down BMPR2 or/and Beclin-1 expression in cells. Their sequences were as follows: sense strand siBMPR2 no. 1 (siBMPR2) primer, 5′-ATTAGACUUUACCUAUUAUGAUGCU-3′ and antisense strand siBMPR2 no. 1 (siBMPR2) primer, 5′-AGCAUCAUAAUAGGUAAAGUCUAAT-3′ sense strand siBMPR2 no. 2 primer, 5′-GAGACUUAAGUGUUCAUUGAGAGGC-3′ and antisense strand siBMPR2 no. 2 primer, 5′-GCCUCUCAAUGAACACUUAAGUCTC-3′ sense strand siBMPR2 no. 3 primer, 5′-ATTAGACUUUACCUAUUAUGAUGCT-3′ and antisense strand siBMPR2 no. 3 primer, 5′-AGCAUCAUAAUAGGUAAAGUCUAAT-3′ sense strand siXIAP primer, 5′-GTGGTAGTCCTGTTTCAGC-3′ and antisense strand siXIAP primer, 5′- GCTGAAACAGGACTACCAC-3′ strand siBeclin-1 primer, 5′-AAGAUCCUGGACCGGGUCACC-3′ and antisense strand siBeclin-1 primer, 5′-GGUGACCCGGUCCAGGAUCTT-3′. The nonspecific siRNAs used as control were following: forward 5′-UUCUCCGAACGUGUCACGUTT-3′ and reverse 5′-ACGUGACACGUUCGGAGAATT-3′. For transfection, cells were grown to ~60% confluence and then transfected with the siRNAs using the Lipofectamine 2000 Kit (Origene, Rockville, MD, USA), according to the manufacturer's instructions.

One day before the experiment, cells were suspended in 200 *μ*l of medium in a 96-well plate at a concentration of 5000 cells per well. Cell viability was assessed by MTS (3-(4,5-dimethylthiazol-2-yl)-5-(3-carboxymethoxyphenyl)-2-(4-sulfophenyl)-2*H*-tetrazolium) assay as described previously.^42^

For colony formation assay, cells, trypsinized during the exponential phase of cell growth, were subcultured into six-well plates at a destiny of 1000 cells per well. After 7 days colonizing, cells were fixed in methyl alcohol for 10 min and stained with crystal violet staining solution.

### Flow cytometry assay

Apoptosis was detected and assayed by an Annexin-V/FITC Kit (BD Biosciences, San Jose, CA, USA) according to the manufacturer's instructions and analyzed by flow cytometry after a series of processes as described previously.^42^

For cell cycle assay, cells were trypsinized and collected in PBS via centrifugation, fixed in 70% ethanol, permeabilized in 0.2% Triton X-100, digested with RNAse A and then stained by PI. The population of nuclei in each phase of the cell cycle was determined and analyzed as described previously.^42^

### Western blot assay

Standard western blot assays were performed as described previously.^42^ Anti-human BMPR2 (ab78422) and total-Smad1/5 (ab75273) antibodies were purchased from Abcam (Cambridge, UK). LC3 (L7543) antibodies were purchased from Sigma (St. Louis, MO, USA). Antibodies against PARP (no. 9542), caspase-3 (no. 9662), XIAP (no. 2042), p53 (no. 2527S), p-Smad1/5 (no. 9516), cyclin B1 (no. 4135), p-Rb (no. 3590), Rb (no. 9309), p62 (no. 5114) and Beclin-1 (no. 3495) were purchased from Cell Signaling Technology (Beverly, MA, USA). Anti-human Mdm2 (sc-812) and *β*-actin (sc-130301) were purchased from Santa Cruz Biotechnology (Santa Cruz, CA, USA).

### Immunofluorescence assay

For immunofluorescence assay of LC3, cells were grown on coverslips and transfected with the BMPR2 siRNA, and incubated with rabbit polyclonal anti-LC3 Ab (1 : 200) overnight at 4 °C. Cells were washed three times with PBS and then reacted with anti-rabbit IgG conjugated with Dylight 488 (1 : 400) for 1 h at room temperature. After washing with PBS, the cells were viewed using confocal microscopy (Leica, Heidelberg, Germany).^43, 44^

### Transmission electron microscopy

After 48 h of BMPR2 siRNA treatment, TEM assay was performed on cells. For TEM assay, cells were digested with 0.25% trypsin and suspended at a concentration of 1.0 × 10^6^ per ml and fixation was carried out at 4 °C for 3 h with 3% glutaraldehyde. Later, ultrathin sections (100 nm) were prepared, stained with uranyl acetate and lead citrate and examined under an electron transmission microscope (H-600; Hitachi, Tokyo, Japan).

### Xenograft tumorigenicity assays

Six-week-old BALB/c female athymic nude mice (Vitalriver, Beijing, China) were subcutaneously injected in the right flank with cells (2 × 10^6^ in 0.1 ml PBS). Once chondrosarcoma cells developed palpable tumors, the mice were randomly divided into two groups for daily intratumoral injection of siBMPR2 or siNC for 4 weeks. For each injection, 5 *μ*g BMPR2 siRNA or negative control siRNA was mixed with 8 *μ*l transfection reagent (Entranster-*in vivo*; Engreen, Beijing, China) *in vivo*, and then to achieve the desired dose were hydrated with 10% glucose injection at a concentration of 50 *μ*g/ml, in accordance with the manufacturer's instructions. After injection, mice were examined and tumor volumes were measured once every 3 days for 4 weeks using a caliper (tumor volume=(length × width^2^)/2). Tumor samples were processed for routine western blot assay.

### Statistical analysis

All statistical analyses were performed using the SPSS19.0 software package (SPSS Inc., Chicago, IL, USA). The relationship between BMPR2 and the survival of patients was evaluated by the Kaplan–Meier analysis, and the relationship between BMPR2 and clinicopathological variables was analyzed by standard *χ*^2^ test. The differences were assessed by the log-rank test and the data were analyzed by one-way analysis of variance with the multiple comparison test of Bonferroni. Student's *t*-tests were used for comparison between two groups. Data are represented as mean±S.D. A value of *P*<0.05 was considered statistically significant.

## Figures and Tables

**Table 1:** The relationship between BMPR2 expression and clinicopathological variables of chondrosarcoma

Clinicopathological variables	No. of cases	BMPR2 expression		*P*-value
		Negative	Positive	
*Sex*				0.359
Male	45	20	25	
Female	33	19	14	
				
*Age (years)*				0.810
≥40	52	25	27	
<40	26	14	12	
				
*Anatomical location*				0.238
Limb bone	28	17	11	
Axial bone	50	22	28	
				
*Grade of tumor*				0.002
Low (grade I)	25	18	7	
High (grade II+III)	30	16	14	
Dedifferentiated	23	5	18	

## Abbreviations

BMP: bone morphogenetic protein
BMPR: bone morphogenetic protein receptor
BMPR2: bone morphogenetic protein receptor 2
siRNA: small interfering RNA
siBMPR2: BMPR2 small interfering RNA
siNC: small interfering RNA negative control
XIAP: X-linked inhibitor of apoptosis
IHC: immunohistochemistry
TEM: transmission electron microscopy
3-MA: 3-methyladenine
LC3: microtubule-associated protein light chain 3
